# The Medical Society of the State of New York

**Published:** 1892-03

**Authors:** 


### BUFFALO MEDICAL AND SURGICAL JOURNAL

A MONTHLY REVIEW OF MEDICINE AND SURGERY.

*EDITORS:*

THOMAS LOTHROP, M. D. - - WM. WARREN POTTER, M. D.

All communications, whether of a literary or business character, should be addressed to the managing editor: 284 Franklin Street, Buffalo, N. Y.

Vol. XXXI. MARCH, 1892. No. 8.

### THE MEDICAL SOCIETY OF THE STATE OF NEW YORK.

The eighty-sixth annual meeting of this Society, which was held in Albany, February 2, 3, and 4, 1892, was in all respects an interesting and profitable gathering. The meetings of this Society have increased in interest very much during late years, and the recent one has kept pace with a large group of its predecessors in respect to number in attendance, value of the scientific work done, and agreeableness of its social entertainments.

The President of the Society, Dr. A. Walter Suiter, of Herkimer, acquitted himself with great credit as an executive officer, and it must be confessed that his inaugural and anniversary addresses were among the best ever heard from the chair of this Society. The inaugural showed great familiarity with the affairs of the profession in this State, and it dealt with all questions of medical polity with much acumen and rare discretion.

The Pan-American Medical Congress received due attention, and the recommendations of the President were indorsed by the Society creating a committee of seven to carry out any regulations imposed upon the Society by the officers of the Congress and to place the Society in every way *en rapport* with the Congress.

Dr. Samuel B. Ward's dinner, on Monday evening, to the ex-presidents of the Society, was a delightful gathering of genial men, who passed three or four hours together in a most agreeable manner.

It has come to pass, of late years, that the Society really begins its meetings on Monday evening, when many of the preliminaries are arranged, and at which time the credentials committee commences its work. This is wise, for it relieves the pressure on this committee in the early hours of Tuesday.

Much disappointment was expressed at the failure to attend of several prominent men from the West and South-west, whose names were on the programme.

The scientific part of the meeting reached its climax on Wednesday afternoon, when the paper of Dr. Wylie was read, and the interest finally culminated in the brilliant discussion that followed Dr. Mordecai Price's paper on Drainage in Abdominal Work, which lasted until 6.30 P. M.

Considerable interest was felt on the subject of the State Examining and Licensing Board, and a brief report was made with reference to its work during the current year. A special committee was appointed by the Society to attend the hearing on a so-called Students' Exemption Bill, introduced by Senator O'Connor, and known as Senate Bill No. 145. We understand that certain students have employed a "professional legislator," who has agreed to obtain their exemption from the provisions of the State Examiners' law for the sum of $5,000 ; or, failing to accomplish this, he is willing to accept $1,200, or about $400 a month during the term of the Legislature. It seems to be difficult, in the State of New York, to obtain any improvement in reference to the methods of conducting its affairs without encountering the most covert and determined methods of opposition. It is difficult to understand on what grounds of right or justice a demand for further exemption is made, but we shall be perfectly willing to have the law amended, as asked, providing it will direct the word "Exempt" to be stamped in indelible letters across the foreheads as well as the diplomas of every student who is engaged in this nefarious scheme. In this connection we desire to call attention to an editorial in the *New York Medical Journal*, February 13, 1891, which ought to be read by every friend of medical improvement, and ought to be committed to heart by every student who is seeking an exemption. We indorse what the accomplished editor of the journal referred to has written on this subject, and commend it to the legislative and executive authorities at Albany.

The Society elected the following officers for the ensuing year: *President*, Dr. Lewis S. Pilcher, of Brooklyn ; *Vice-President*, Dr. H. L. Elsner, of Syracuse, N. Y.; *Secretary*, Dr. F. C. Curtis, of Albany ; *Treasurer*, Dr. C. H. Porter, of Albany. Standing Committees: On *Arrangements*, Herman Bendel, Seneca D. Powell, and Nellis ; *By-Laws*, A. R. Simmons, W. C. Wey, and Curtis ; *Hygiene*, Laurence Johnson, Brush, Bell, Burr, Balch, Herman, and

Peck; *Legislation*, D. B. St. John Roosa, Daniel Lewis, and Lewi; *Ethics*, A. Jacobi, A. Matheson, and J. H. Glass; *Prize Essays*, Geo. H. Fox, W. W. Potter, and Roe; *Publication*, Curtis, Potter, Porter, and Bailey. The following named were elected *Honorary Members*: Dr. Lewis S. McMurtry, of Louisville, Ky.; Dr. Charles A. L. Reed, of Cincinnati, O.; Dr. William E. B. Davis, of Rome, Ga.; Dr. Adam H. Wright, and Dr. James F. W. Ross, of Toronto, Ont.

